# Bayesian forecasting of disease spread with little or no local data

**DOI:** 10.1038/s41598-023-35177-6

**Published:** 2023-05-19

**Authors:** Jonathan D. Cook, David M. Williams, Daniel P. Walsh, Trevor J. Hefley

**Affiliations:** 1 Michigan State University, 480 Wilson Road, East Lansing, MI 48823 USA; 2 U.S. Geological Survey, Montana Cooperative Wildlife Research Unit, University of Montana, 32 Campus Drive NS 205, Missoula, MT 59812 USA; 3 Department of Statistics, Kansas State University, 1116 Mid-Campus Drive N, Manhattan, KS 66506 USA

**Keywords:** Ecological modelling, Ecological epidemiology, Statistics

## Abstract

Rapid and targeted management actions are a prerequisite to efficiently mitigate disease outbreaks. Targeted actions, however, require accurate spatial information on disease occurrence and spread. Frequently, targeted management actions are guided by non-statistical approaches that define the affected area by a pre-determined distance surrounding a small number of disease detections. As an alternative, we present a long-recognized but underutilized Bayesian technique that uses limited local data and informative priors to make statistically valid predictions and forecasts about disease occurrence and spread. As a case study, we use limited local data that were available after the detection of chronic wasting disease in Michigan, U.S. along with information rich priors obtained from a previous study in a neighboring state. Using these limited local data and informative priors, we generate statistically valid predictions of disease occurrence and spread for the Michigan study area. This Bayesian technique is conceptually and computationally simple, relies on little to no local data, and is competitive with non-statistical distance-based metrics in all performance evaluations. Bayesian modeling has added benefits because it allows practitioners to generate immediate forecasts of future disease conditions and provides a principled framework to incorporate new data as they accumulate. We contend that the Bayesian technique offers broad-scale benefits and opportunities to make statistical inference across a diversity of data-deficient systems, not limited to disease.

## Introduction

Mitigating the effects of disease on affected populations is best achieved by enacting rapid and targeted actions immediately upon first detection^1,2^. However, to efficiently guide these early targeted actions, management agencies need accurate spatial information on where disease occurs and how it might be spreading and changing in intensity. Management agencies would further benefit from a formalized way to incorporate new data as they perform disease surveillance in response to these new detections. Many scientific fields, including ecology and epidemiology, have adopted statistical modelling as the main tool used to obtain inference, predictions, and forecasts for dynamic spatio-temporal processes like disease outbreaks. Yet, statistical approaches are often considered unfeasible following a recent detection of an emerging disease because of limited local data.

As an alternative to statistical approaches, agencies tasked with managing diseases have adopted non-statistical approaches, such as encircling disease detections by a pre-determined distance (hereafter “rule-based” approach). Simple rule-based approaches define where actions should be applied uniformly and surround the locations of detection based on the ecology, contact structure, and movement patterns of affected hosts. The continued use of distance buffers across many years and jurisdictions suggests that agencies are generally satisfied with their performance; however, these buffers do not provide spatially-explicit estimates of disease spread and growth and thus may not fully leverage existing information to improve targeted actions, resulting in potential suboptimal application of disease management efforts and resource use. Distance buffers further lack the ability to learn from new data as it accumulates to forecast future disease conditions. Thus, there is an opportunity to improve the spatial resolution of information available to managers upon first detection by leveraging existing information and limited sources of data to generate probabilistic predictions and forecasts of disease spread and growth.

Bayesian statistical models have been used in situations where local data lack sufficient information to fit models, but where other sources of existing information can supplement unknown parameters^3^. In fact, Bayesian statistical models are particularly well-suited for the problem of prediction and forecasting with little or no data. For newly detected diseases, Bayesian statistical models may be a particularly powerful approach. For example, probabilistic predictions from Bayesian statistical models can be used to delineate the spatial extent and occurrence of disease. These spatial predictions can also be forecasted into the future to understand disease spread and growth which provides the opportunity for targeted proactive management. Bayesian statistical models can also readily incorporate (be fit to) new data and updated results can be used to plan future actions.

We evaluate the potential strengths of Bayesian modeling with limited local data by estimating the spatial extent and occurrence of chronic wasting disease (CWD) in white-tailed deer (*Odocoileus virginianus*) in Michigan, U.S. using only information and limited local data that were available immediately following initial detections. We then compare the performance of our Bayesian predictions (hereafter “Bayesian” approach) against an existing rule-based approach. While our application is focused on CWD, Bayesian modeling can be extended to make statistically principled predictions and forecasts of dynamic spatio-temporal processes across a variety of data-deficient systems. Potential applications of Bayesian modeling without local data include evaluations of invasive species introduction and spread and species status assessments for species of conservation concern.

### The Bayesian approach with little or no local data

There are three main information rich components required to obtain probabilistic predictions and forecasts from a Bayesian model when local data are limited:

1. At least one dynamic spatio-temporal model that is appropriate to the system (see Wikle et al.^4^ Ch. 5),
2. Relevant posterior distributions, obtained from a similar system, for as many of the parameters for the model chosen in step 1 as possible,
3. A small amount of data or expert knowledge that can be used as priors for any model parameters where posterior distributions from 2 are lacking.

After these three sources of information are gathered, obtaining probabilistic predictions and forecasts is automatic via the prior predictive distribution which we explain in the next sub-section. We use standard notation from Bayesian statistics throughout, such that a square bracket represents a probability distribution (e.g., $[a]$ where *a* is the random variable), and a vertical bar inside of the bracket means that the probability distribution is conditional on any variable following it within the bracket (e.g., $[a|b]$ where *a* is the random variable conditional on *b*)^5^.

### The prior predictive distribution

Bayes rule states that

1 $$\left[{\varvec{\theta}}|\mathbf{y}\right]=\frac{\left[\mathbf{y}|{\varvec{\theta}}\right][{\varvec{\theta}}]}{\left[\mathbf{y}\right]} ,$$

where ${\varvec{\theta}}$ is a vector of model parameters and $\mathbf{y}$ is a vector of data. The well-known constituent parts of Bayes rule include the posterior distribution ($[{\varvec{\theta}}|\mathbf{y}]$), the model or likelihood ($[\mathbf{y}|{\varvec{\theta}}]$), and the prior ($[{\varvec{\theta}}]$). However, there is also a lesser-known component of Bayes rule that is the normalizing constant $[\mathbf{y}]$. The normalizing constant is obtained by integrating the product of the likelihood and the prior as shown:

2 $$\left[\mathbf{y}\right]=\int \left[\mathbf{y}|{\varvec{\theta}}\right]\left[{\varvec{\theta}}\right]d{\varvec{\theta}}.$$

When fitting a Bayesian model to data, the distribution $[\mathbf{y}]$ is a mathematical function that depends solely on data and outputs a single number once the data are “plugged into” that function. This single number ensures that the posterior distribution ($[{\varvec{\theta}}|\mathbf{y}]$) integrates to one, hence the unflattering moniker for $[\mathbf{y}]$, the normalizing constant.

In situations where data are sufficient for model fitting and estimation, the normalizing constant garners little attention outside of the literature on Bayesian computing and model checking. For example, modern Bayesian model fitting algorithms such as Markov chain Monte Carlo (MCMC) are designed to side-step the calculation of $[\mathbf{y}]$ entirely (see Hooten and Hobbs^5^ Ch. 7), whereas Bayesian model-checking practices occasionally use $[\mathbf{y}]$ where it has been called the prior predictive distribution^6^. Model checking based on the prior predictive distribution has been even less popular and thus, well known, when compared to approaches that use the posterior predictive distributions^6^.

In the absence of data, however, the prior predictive distribution is a powerful and underutilized part of Bayes statistics that can be used to make inference (e.g., Dexter and Ledolter^3^). Given a specified model (Component 1 above), $[\mathbf{y}|{\varvec{\theta}}]$, which includes both a deterministic (e.g., a mathematical model) and stochastic component (e.g., a data generating probability distribution; see Hooten and Hobbs^5^ Ch. 4), we can use information rich priors from Component 2 or 3 above with the prior predictive distribution to make inference, predictions, and forecasts without data. In the next sub-section, we explain a simple computational algorithm to obtain samples from the prior predictive distribution similar in kind to well-known approaches used to fit Bayesian models to data.

### Model implementation

For most Bayesian models, obtaining the prior predictive distribution will not be possible analytically. However, with the rise in Bayesian statistical methods, Bayesian model fitting approaches, such as MCMC, have become almost as commonplace as traditional fitting approaches like least squares. As such, what follows relies on understanding Monte Carlo based approaches for Bayesian model fitting. For additional background, we refer the reader to ecology-focused references, such as Hooten and Hobbs^5^.

When suitable data are not available to fit a Bayesian model to data, composition sampling^7^ (see Hooten and Hobbs^5^ pp. 196–201) and derived quantities (Hooten and Hobbs^5^ pp. 194–196) can be used. Composition sampling is a Monte Carlo approach that is considerably easier to implement when compared to Bayesian model fitting approaches such as MCMC and is applied by first obtaining a draw from the prior distribution(s) (i.e., $[{\varvec{\theta}}]$). The draw from each prior is then “plugged into” the model and a stochastic prediction or forecast is made by taking a draw from the model (i.e., $[\mathbf{y}|{\varvec{\theta}}]$). The steps are then repeated many times to obtain the same number of draws from the prior predictive distribution (i.e., $[\mathbf{y}]$). The samples for any given prediction or forecast, such as the disease occurrence at a particular location at some future point in time, can then be summarized as derived quantities (i.e., functions of the prior predictive distribution such as the mean or median).

### Application of Bayesian modeling to chronic wasting disease in Michigan

To illustrate the power of Bayes statistics to make inference in data deficient systems, we make spatial predictions and forecasts of the probability of infection (i.e., probability of CWD occurrence) for white-tailed deer in Michigan, U.S., following a recent detection in 2017. Chronic wasting disease is a fatal neurodegenerative prion disease that affects cervids. It has repeatedly emerged and spread in cervid species across North America and has occurred in other locations such as Norway, Finland, and South Korea^8^. The repeated emergence of CWD at different locations highlights an opportunity for valid statistical approaches to estimate the intensity and spatial extent of disease in areas of recent detection so that management actions can be implemented.

**Figure 1:**
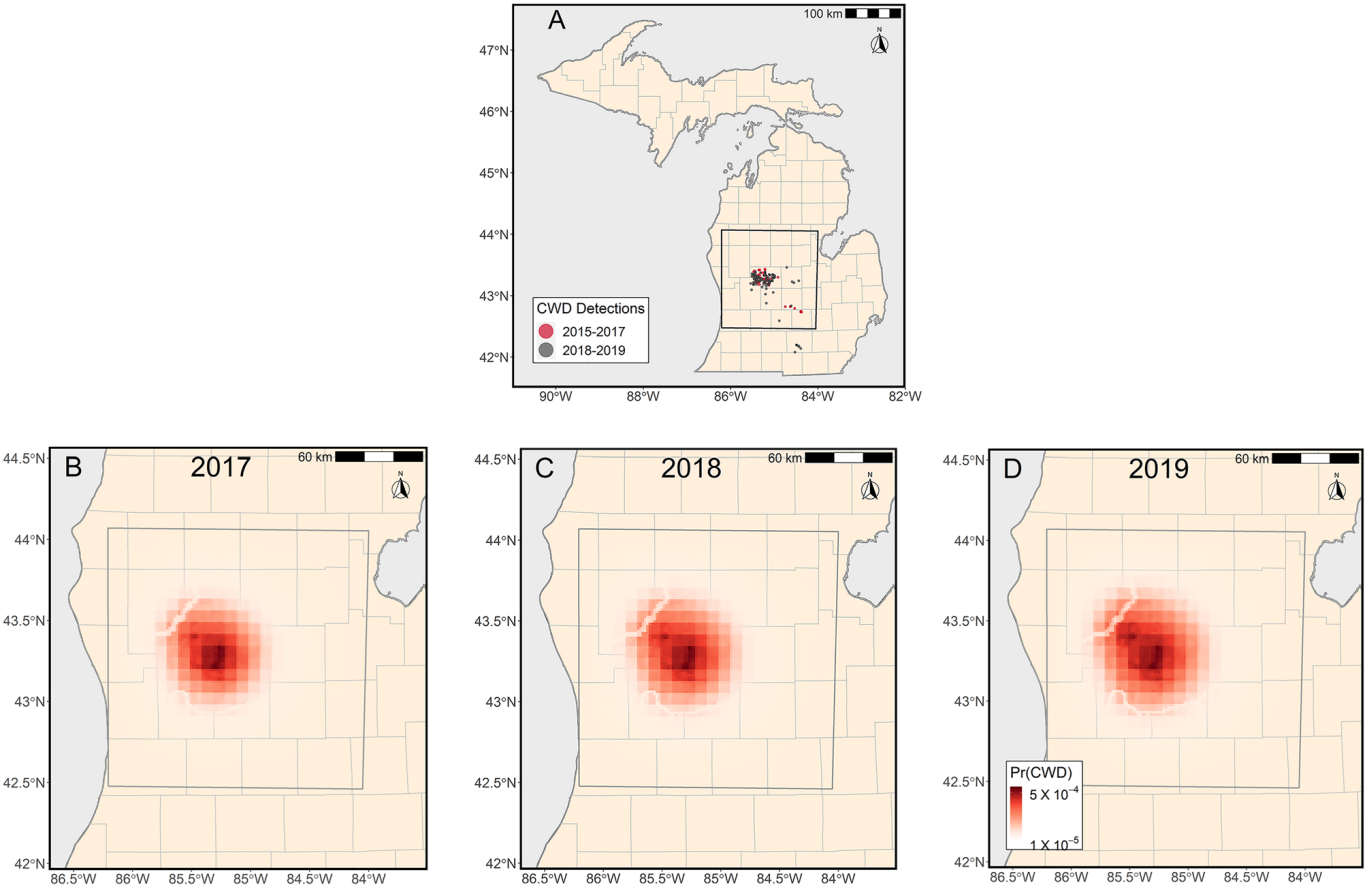
(**A**–**D**) (**A**) is a map of CWD positives detected in Michigan from 2015 to 2019. The red symbols indicate the 54 total positive detections from 2015 to 2017 within our study area (black box), of which 45 were used in model projection (the detections in Clinton and Ingham were excluded). The black symbols indicate the 117 positive detections from 2018 to 2019 that were used for model performance evaluations. Nine additional positives from 2018 to 2019 fell outside of our study area. (**B**–**D**) are the expected value of the probability of infection and spatial extent for adult male white-tailed deer across three years. Panel B is 2017, Panel C is 2018 and Panel D is 2019. Maps were created in Program R version 3.5.2 (R Core Team 2018) using results of our analyses and publicly owned data from the state of Michigan, U.S. (https://gis-michigan.opendata.arcgis.com/).

In 2017, the Michigan Department of Natural Resources (MDNR) detected CWD in a 3-year-old female white-tailed deer submitted during an early season youth hunt in Montcalm County^9^. Additional surveillance in the area identified 45 total CWD-positive animals in a concentrated disease cluster that were geographically distant from prior positives in 2015 and 2016 (Fig. 1A). Based on that single year of limited observation, it was perceived that no data or knowledge were available to provide statistical inference on local disease occurrence including its spatiotemporal spread and growth. Thus, a standardized rule-based approach was perceived to be the only existing method available to produce an estimate of disease extent.

We wanted to evaluate whether we could use Bayesian modeling to enhance the information available immediately following the detection in Michigan. We used an existing model developed in a nearby state (Component 1 above), relevant posterior distributions estimated using 14 years of CWD monitoring data from a nearby state (Component 2 above), and a small amount of local data (Component 3 above) to make current-year predictions and forecasts for future years. We compared the performance of our predictions and forecasts against the existing rule-based approach to determine whether Bayesian modeling enhanced our understanding of disease dynamics immediately upon first detection and provided opportunity for more informed disease management activities.

## Results

### Bayesian modeling of disease spread with few local data

Using the existing Bayesian model and posteriors, along with limited surveillance data from Michigan, we made statistical inference across space and time in a manner not possible with rule-based approaches. We found that the expected value for the probability of CWD infection varied across our study area (Fig. 1A) and ranged from a minimum of 0 in the boundary regions up to a maximum of 0.0028 in central locations where the majority of CWD detections were identified in 2018 and 2019 (Fig. 1B–D). In terms of temporal variation, the centrally located areas with elevated expected values in 2017 tended to increase in magnitude and extent when compared against 2019 forecasts in a manner that, again, matched the distribution of CWD detections from 2018 and 2019 (Fig. 1B–D). In contrast, the rule-based approach provided no such probability gradient for visual assessment, or the ability to forecast CWD spread patterns into future years.

### Comparison of Bayesian modeling to a common rule-based approach

**Figure 2:**
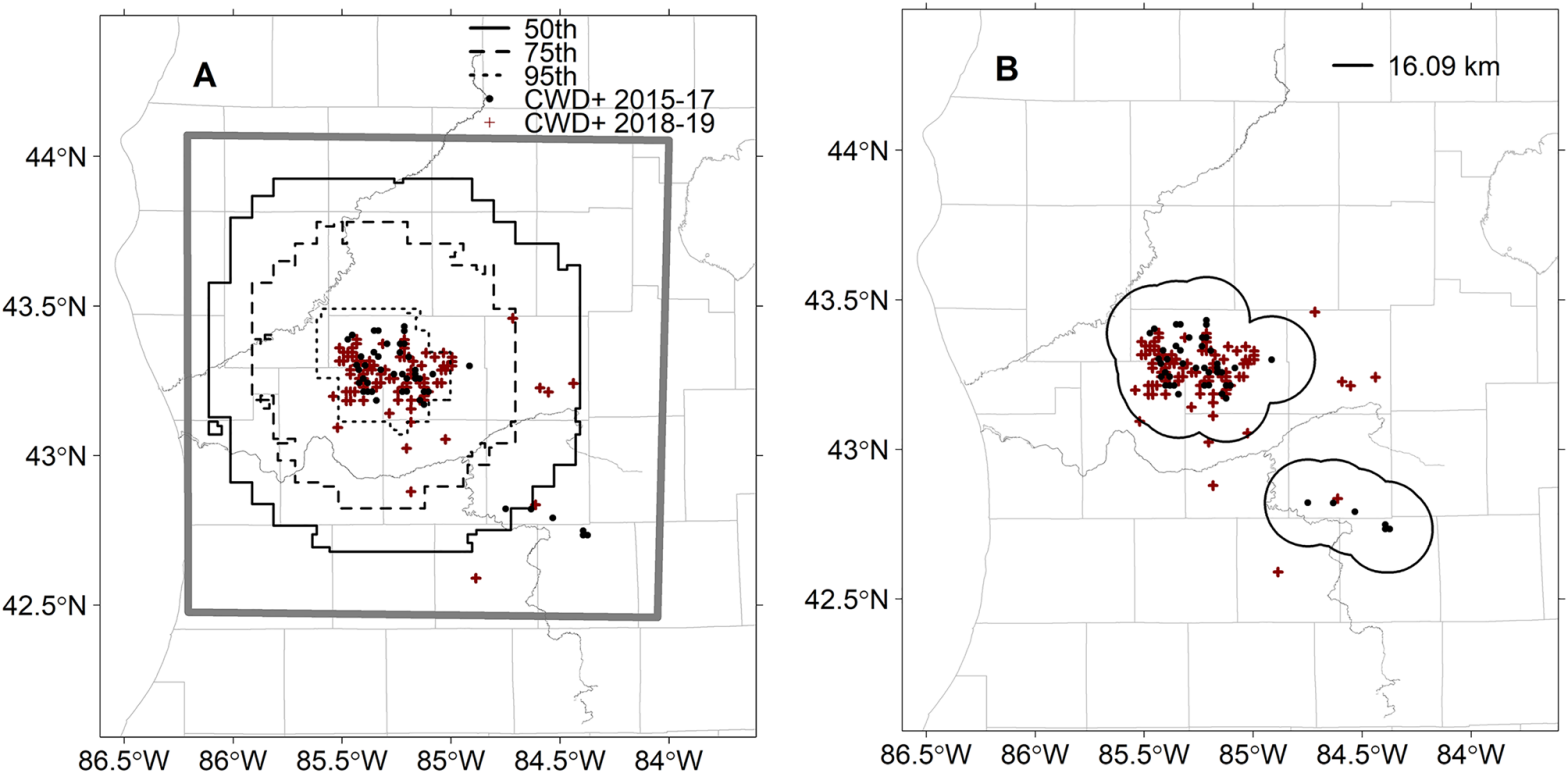
(**A**,**B**) (**A**) depicts the three probabilistic disease extent estimates generated from 50th, 75th, and 95th percentiles using the expected value of the probability of infection from 2017. (**B**) is a disease extent estimate based on a 16.09 km buffer surrounding 2015–2017 positive detections (black line). Grey box is the extent of our study area. *Note* Higher percentile values are smaller in area because they exclude values below the respective percentile point estimates, e.g., 95th percentile cutoff excludes more probability of infection values than a 50th percentile.

For direct model comparison, we found that our Bayesian model forecasts performed better and were able to encompass more future disease detections when compared against the rule-based approach. The Bernoulli Deviance estimate was 1544.51 for the Bayesian model and 1574.96 for the rule-based approach. Further, we found that disease extent estimates derived from expected value percentiles were able to include more future positives than the rule-based approach. In terms of total area, the smallest CWD extent based on 2017 projections covered a total area of 1.57 × 10^3^ km^2^ using a 95th percentile cutoff, followed by the 75th (total area: 7.87 × 10^3^ km^2^) and 50th percentile (total area: 1.57 × 10^4^ km^2^) (Fig. 1D). (Note: Higher percentile values are smaller in area because they exclude values below the respective percentile point estimates, e.g., 95th percentile cutoff excludes more probability of occurrence values than a 50th percentile cutoff). Compared with a rule-based disease extent estimate based on the location of 2017 detections (total area: 5.30 × 10^3^ km^2^; Fig. 2B), the 75th and 50th percentiles were larger in total area, whereas the 95th percentile cutoff was smaller (Fig. 2A,B). The 95th percentiles encompassed 101/117 (86% of) CWD detections from 2018 and 2019, whereas the 75th and 50th percentiles encompassed 112 (96%) and 115 (98%) of 117 CWD detections, respectively. The rule-based extent encompassed 109/117 CWD detections. Most notably for disease extent, the rule-based approach underestimated the local extent of CWD in the area immediately surrounding the cluster first detected in 2017.

## Discussion

Broadly, our study shows how Bayes statistics can be used to make inference, predictions, and forecasts in a data-deficient but information-rich disease system. We relied on a long-recognized statistical technique of composition sampling^7^ using Monte Carlo simulation to produce samples from the prior predictive distribution and derived quantities from an existing spatio-temporal model of CWD dynamics. We used available information to make statistical inference, including limited data from Michigan, U.S. and posteriors estimated from a CWD study on the same species in the same ecoregion^10^. Our results were competitive with an existing rule-based approach and provided a source of reliable and rapid information to guide targeted management actions immediately upon the first detection of CWD in a new area. While it is desirable for the Bayesian approach to compete with the existing rule-based method, we believe that the greatest benefits of statistical inference are its advantages of providing statistically based forecasts and a principled framework to “learn” from new data. However, as with any statistical model or rule-based approach, the relative performance of the two methods depends on the assumptions. In our case, the relative performance of the two methods, depends on the distribution of disease cases observed in 2018 and 2019 as well as the values we used for disease probability in the rule-based implementation.

When compared against existing rule-based approaches, our Bayesian approach offered at least two notable advantages that likely transcend to other data-deficient systems. First, we made inference on the spatio-temporal dynamics of disease occurrence that was specific to the composition and configuration of Michigan’s landscape with only a single year of data. We then used derived quantities to predict and forecast disease dynamics in current and future years in a manner that directly informs applied and rapid management efforts at locations at risk. In contrast, existing rule-based approaches provide no such opportunity. Second, Bayesian-models provided an opportunity to learn from current and future disease observations and thereby allow managers to update their knowledge about disease spread and growth dynamics in real time as new data accumulate. They can further use this updated knowledge as feedback to guide decisions on how to respond to changes in disease condition over time.

One additional benefit, while not necessary to justify the use of Bayesian modeling, is that the results were immediately competitive with rule-based approach in an area that is presumably its strength—encompassing future cases of disease. Several of our disease extent estimates using our Bayesian model encompassed more future positives and were less prone to underestimation when compared against a rule-based approach. While larger extents seemingly require more resources to manage, strategic expansions of disease-affected areas combined with statistical inference on which areas to target may result in an overall reduction in resources necessary to mount precision management^12^. Thus, Bayesian modeling can provide statistically valid and defensible justification for expanding precision control efforts into areas where disease likely exists but is not addressed because of a lack of spatial inference provided by rule-based approaches.

The higher performance for the Bayesian approach indicated that forecasts of CWD spread and growth estimated using informative priors from Wisconsin and with little local Michigan data accurately characterized the gradient in probability of infection. However, one caveat of Bayesian forecasting without local data is its reliance on informative priors. Care needs to be taken to select models and priors that are representative of the system, and to ensure that parametric uncertainty is fully considered^3^. Potential options to develop informative priors include the development of global estimates of relevant parameters that include variation across areas with sufficient data for local estimation, or by using other reliable methods for parameter estimation without empirical data, such as expert elicitation. We made other assumptions about the Michigan CWD detections that informed our choice of priors and model implementation. Two notable assumptions were that CWD had been on the landscape for a time prior to detection and that introduction occurred at a single point and spread outwardly. These assumptions would be worth revisiting as more surveillance data are collected in Michigan over time.

Our illustrated Bayesian approach addresses a fundamental need universal to wildlife disease managers—the need to estimate affected area early upon emergence, or detection of disease in a new area or host using limited data^18^. For diseases warranting active interventions, such as targeted removals, Bayesian methods can also be used to provide spatio-temporal estimates of risk that guide precision management efforts. The ability for targeted removal of animals at locations of high risk may ultimately improve the efficiency and effectiveness of disease interventions. Rule-based approaches remain useful, such as in cases where information regarding disease process is lacking; however, using methods such as ours that incorporate local information (e.g., landscape configuration, number of positive detections) have the potential to greatly improve disease management efforts across a diversity of wildlife diseases. Our approach is useful to clustering diseases, such as bovine tuberculosis, rabies, and avian influenza, as well as other data-deficient ecological systems that can be modeled using a dynamic spatio-temporal model and for which posterior estimates of model parameters can be drawn from past research. Beyond diseases, we expect broad applications and benefits of our work, such as invasive plant or animal species, or status assessments for imperiled species without enough data to fit models.

## Methods

For our model (component 1), we used a slightly modified version of the model from Hefley et al.^10^ which is:

3 $${y}_{i}\sim Bernoulli\left({p}_{i}\right),$$

4 $$g\left({p}_{i}\right)=u\left({\mathbf{s}}_{i},{t}_{i}\right){e}^{{\mathbf{x}}_{i}^{^{\prime}}{\varvec{\beta}}},$$

5 $$\frac{\partial }{{\partial }_{t}}u\left(\mathbf{s},t\right)=\left(\frac{{\partial }^{2}}{\partial {s}_{1}^{2}}+\frac{{\partial }^{2}}{\partial {s}_{2}^{2}}\right)\mu \left(\mathbf{s}\right)u(\mathbf{s},t)+ \lambda \left(\mathbf{s}\right)u\left(\mathbf{s},t\right),$$

6 $$\mathrm{log}\left(\mu \left(\mathbf{s}\right)\right)= {\alpha }_{0}+{\mathbf{z}(\mathbf{s})}^{^{\prime}}\boldsymbol{\alpha },$$

7 $$\lambda \left(\mathbf{s}\right)= {\gamma }_{0}+{\mathbf{w}(\mathbf{s})}^{^{\prime}}{\varvec{\gamma}}.$$

In Eq. (3), *y*_*i*_ is the disease occurrence of the *ith* deer (i.e., *y*_*i*_ = 1 is positive and *y*_*i*_ = 0 is negative) while *p*_*i*_ is the probability of occurrence. In Eq. (4), $u\left({\mathbf{s}}_{i},{t}_{i}\right)$ is the intensity at the coordinates ${\mathbf{s}}_{i}$ and time ${t}_{i}$ that is scaled by ${e}^{{\mathbf{x}}_{i}^{^{\prime}}{\varvec{\beta}}}$ and transformed into a probability by the inverse of the link function $g\left(\cdot \right)$. As in Hefley et al.^10^, the link function is a truncated cumulative normal distribution. Also, in Eq. (4) are individual-level (i.e., non-spatial) covariates, **x**_*i*_, including the sex and age of tested deer, that scale the intensity via the parameters contained within the vector ${\varvec{\beta}}$. The intensity $u\left(\mathbf{s},t\right)$ varies over space and time according to the partial differential equation (PDE) in Eq. (5). As in Hefley et al.^10^, the diffusion, $\mu \left(\mathbf{s}\right)$, and growth rate, $\lambda \left(\mathbf{s}\right)$, of the PDE in Eq. (5) depend on spatial covariates such as landscape characteristics. Thus, the PDE provides an efficient and continuous formulation of the mechanistic linkage between temporal processes like disease growth with transient spatial processes, such as variation in habitat types across a study area. We chose covariates that were consistent with Hefley et al.^10^ and included proportion hardwood forest, human development, and large river corridors modeled using regression-type equations in Eqs. (6) and (7), respectively, and adult male deer as the reference class in Eq. (4). The vectors $\mathbf{z}\left(\mathbf{s}\right)$ and $\mathbf{w}\left(\mathbf{s}\right)$ contain the spatial information for the landscape covariates used in the diffusion and growth Eqs. (6 and 7). The parameters ${\alpha }_{0}$ and $\boldsymbol{\alpha }$ control the diffusion rate in Eq. (6), and ${\gamma }_{0}$ and ${\varvec{\gamma}}$ control the growth rate in Eq. (7). Finally, “solving” the PDE in Eq. (5) required that initial conditions for space and time were provided. For initial conditions, we used

8 $$u(\mathbf{s},t)=\left\{\begin{array}{cc}\theta & \mathrm{if}\ \mathbf{s}={\varvec{\upomega}}\ \mathrm{and}\ t = \tau \\ 0& \mathrm{if}\ \mathbf{s}\ne {\varvec{\upomega}} \boldsymbol{ }\ \mathrm{or}\ t \ne \tau \end{array},\right.$$

where $\tau$ is a parameter that represents the time, measured in years prior to 2017, that CWD was introduced into the Michigan study area. For the location of introduction, ${\varvec{\upomega}}$ is a vector of two parameters which represents the coordinates (i.e., location) where CWD was introduced. The parameter $\theta$ is the initial intensity. We also assumed that all cells at the boundary were equal to zero, i.e., $u\left(\mathbf{s},t\right)=0$, for all *t.* For a more complete explanation of the model, including further description of how the PDE was implemented, we refer the reader to Hefley et al.^10^ and specifically Appendix S1.

### Priors for CWD model in Michigan

For the parameters ${\varvec{\beta}}$, ${\alpha }_{0}$, $\boldsymbol{\alpha }$, ${\gamma }_{0}$, and ${\varvec{\gamma}}$, we specified priors by assuming independent normal distributions for each parameter. The distributions had an expected value and variance based on those reported in Hefley et al.^10^ (Supplementary Table S1.1). To specify priors for initial conditions relevant to Michigan (i.e., Eq. 8), we used all available information and data, including the locations of 2017 CWD detections and historical knowledge of CWD detection in both free-ranging and captive white-tailed deer. Since we are not fitting the Bayesian model in Eqs. (3–8) to data, we used these limited data to develop informative priors for ${\varvec{\upomega}}$ and $\tau .$

For the location where CWD was introduced in Michigan, ${\varvec{\upomega}}$, we specified a prior using all CWD positive deer from 2017. To translate these limited data into an informative prior, we assumed that the location of introduction was one of the 45 locations where CWD was detected in 2017 (Fig. 1A). More precisely, the informative prior is the probability mass function

9 $${\varvec{\upomega}}\sim \left\{\begin{array}{cc}{\mathbf{q}}_{1}& \mathrm{with}\ \mathrm{probability}\ \frac{1}{45}\\ {\mathbf{q}}_{2}& \mathrm{with}\ \mathrm{probability}\ \frac{1}{45}\\ \vdots & \vdots \\ {\mathbf{q}}_{45}& \mathrm{with}\ \mathrm{probability}\ \frac{1}{45}\end{array}\right.,$$

where ${\mathbf{q}}_{1}, {\mathbf{q}}_{2},...,{\mathbf{q}}_{45}$ are vectors that contain the coordinates of the 45 CWD-positive animals.

To specify the prior for the years since 2017 that CWD was introduced into the Michigan study area, $\tau$, we used knowledge of a CWD detection in a captive white-tailed deer facility from 2008. The facility was located 32 km away from the closest free-ranging positive detected in 2017, and within range of documented deer dispersals^11^. In addition, following the 2008 detection, subsequent sampling in free-ranging deer in 2008 and 2009 surrounding the facility were insufficient to detect CWD at a prevalence below 3.5% (Supplementary Table S1.2; upper 95% credible interval [CI] estimate). Finally, existing knowledge of the slow spread and growth of CWD^12^, combined with the larger number of positives detected in 2017, led us to assume that the time since introduction was associated with the 2008 detection as opposed to more recent detections. Thus, we specified the prior

10 $$\tau \sim \mathrm{Beta}-\mathrm{binomial }(n = 10,\alpha = 1, \beta = 0.5{)}_{1}^{n}$$

where $\mathrm{n}$ is the number of trials, α and β are shape parameters of the beta-binomial distribution such that $E(\tau )= \frac{n\mathrm{\alpha }}{\mathrm{\alpha }+\upbeta }$, and the super- and sub-scripts indicate that the distribution is zero-truncated. The prior specified in Eq. (10) result was a right-skewed prior for $\tau$ that had substantial probability (e.g., 0.05–0.10) placed on values of $\tau$ between 1 and 9 years (prior to 2017) and the highest probability of ~ 0.25 given to $\tau$ of 10 years.

To specify the prior for the initial disease intensity (i.e., $\theta$) we first assumed that CWD was introduced into a single adult male white-tailed deer individual within a single section of land according to the Public Land Survey System. We assumed an adult male because males have been documented as having higher CWD prevalence than females and younger individuals, at least during the early stages of CWD spread in a new location^13^. Because free-ranging white-tailed deer density varies from between 15 and 45 individual deer per square mile (2.6 km^2^) in the agro-forested upper Midwestern U.S.^14^, we assumed an initial probability of infection estimate of

11 $$p\left({\mathbf{s}}^{\boldsymbol{*}},\tau \right) \sim \mathrm{uniform }(\mathrm{min}=\frac{1}{45},\mathrm{ max}=\frac{1}{15})$$

where $p\left({{\mathbf{s}}}^{\boldsymbol{*}},\tau \right)$ is the probability of infection at a square mile location, ${{\mathbf{s}}}^{\boldsymbol{*}}$, and time since introduction,$\tau$. We then rearranged Eq. (4) above as

12 $$\theta =\frac{{g}^{-1}\left(p\left(\mathbf{s}^{\boldsymbol{*}},\tau \right)\right) }{{e}^{{\mathbf{x}}_{i}^{^{\prime}}{\varvec{\beta}}}}.$$

Because the transformations to obtain the prior for $\theta$ cannot be done analytically, we sampled from the prior assigned to $p\left({{\mathbf{s}}}^{\boldsymbol{*}},\tau \right)$ and the posterior distribution for adult male deer in ${e}^{{\mathbf{x}}_{i}^{^{\prime}}{\varvec{\beta}}}$ to obtain the prior for $\theta$ which we then used in our composition sampling approach.

### Derived quantities and model evaluation

We conducted two separate performance evaluations that compared our Bayesian model against an existing rule-based approach. For the rule-based approach, we used a 16.09 km radius surrounding all known positives from 2015 to 2017 to match the distance codified in MDNR CWD response documents^15^. To test the rule-based approach against our model, we used surveillance data collected in our study area after 2017 which included 117 CWD-positive and 32,456 CWD-negative individual deer (Fig. 1A).

The first comparison used a proper scoring rule to evaluate the spatial performance of predictions for the rule-based approach and our Bayesian model projected to year 2017^16^. However, to facilitate a direct comparison between approaches, we were required to make a creative, but fair, assumption that translated the rule-based approach into a probability surface. Because the rule-based approach provided a region surrounding positives where disease was expected to occur and assumed all other areas were free-from-disease, we interpreted it as having a probability of infection inside the boundary equal to the maximum of our Bayesian results from 2018 (0.0029) and 2019 (0.0031) depending on the year of sample collection, whereas all areas outside had a low probability of infection (1 × 10^–6^). Given two probability surfaces, we then used Bernoulli deviance^16^

13 $$-2{\sum }_{j=1}^{J}\left({z}_{j}\mathrm{log}\left({\psi }_{j}\right)+\left(1-{z}_{j}\right)\mathrm{log}\left(1-{\psi }_{j}\right)\right)$$

where ${z}_{j}$ is the disease status of the *jth* deer (i.e., *z*_*j*_ = 1 is positive and *z*_*j*_ = 0 is negative), ${\psi }_{j}$ is the probability of infection (e.g., ${\psi }_{i}\equiv {E(y}_{i})$ for the Bayesian model from Eq. 4) and *J* is the number of individual deer tested after 2017 (i.e., the 117 CWD-positive and 32,456 CWD-negative individuals shown in Fig. 1A). When used as a scoring rule, the deviance is akin to information criteria used in model selection in that lower deviance scores represent better agreement between the data available after 2017 and the Bayesian model or rule-based approach. However, unlike model selection, we aim to determine which technique, either rule-based or our model-based approach, performs best in terms of predictive performance.

The second comparison compared the approaches in a manner that played to the strengths of the rule-based approach. To perform, we used the number of future disease detections from 2018 and 2019 encircled by each approach (Bayesian model or rule-based). Like the first comparison, we transformed one set of results to a comparable condition to the other; however, in this case it was our probabilistic Bayesian model that required transformation to a disease extent. To translate the probabilistic surface into a disease extent estimate, we used the 50th, 75th, and 95th percentiles of the expected value of disease occurrence (i.e., ${E(y}_{i})$) to determine where disease was expected to occur (i.e., all values that exceeded the 50, 75, and 95 percentile point estimates were included inside, and a ringed boundary excluded all values below those percentiles). We then compared the two approaches by evaluating which disease extent estimate best encompassed the 117 CWD detections from 2018 to 2019. All analyses were performed in Program R version 3.5.2^17^.

## Supplementary Information

Supplementary Information.

## Data Availability

The datasets generated and/or analyzed during the current study are available in the Bayesian Composition Sampling repository on GitLab^19^, https://doi.org/10.5066/P9XMF7FS. The dataset that includes the locations of CWD positive white-tailed is owned by Michigan Department of Natural Resources. For access upon reasonable request, please contact Melinda Cosgrove (CosgroveM1@michigan.gov).

## References

[1] Heymann DL (2004). The international response to the outbreak of SARS in 2003. Philos. Trans. R. Soc. Lond. B.

[2] Voyles J, Kilpatrick AM, Collins JP (2014). Moving beyond too little, too late: Managing emerging infectious diseases in wild populations requires international policy and partnerships. EcoHealth.

[3] Dexter F, Ledolter J (2005). Bayesian prediction bounds and comparisons of operating room times even for procedures with few or no historic data. Anesthesiology.

[4] Wikle CK, Zammit-Mangion A, Cressie N (2019). Spatio-Temporal Statistics with R.

[5] Conn PB, Johnson DS, Williams PJ, Melin SR, Hooten MB (2018). A guide to Bayesian model checking for ecologists. Ecol. Monogr..

[6] Tanner MA (1991). Tools for Statistical Inference.

[7] Richards, B. J. *Chronic Wasting Disease Distribution in the United States by State and County: U.S. Geological Survey Data Release*. 10.5066/P9HQKKFO (2021).

[8] Michigan Department of Natural Resources. (MDNR). *Chronic Wasting Disease Response Measures for Deer in Kent and Montcalm Counties. Interim Order Report* 14 (2017).

[9] Hefley TJ, Hooten MB, Russell RE, Walsh DP, Powell JA (2017). When mechanism matters: Bayesian forecasting using models of ecological diffusion. Ecol. Lett..

[10] Nixon CM, Hansen LP, Brewer PA, Chelsvig JE, Sullivan JB, Esker TL, Koerkenmeier R, Etter DR, Cline J, Thomas JA (1994). Behavior, dispersal, and survival of male white-tailed deer in Illinois. Ill. Nat. Hist. Surv. Biol. Notes.

[11] Miller MW, Fischer JR (2016). The first five (or more) decades of chronic wasting disease: Lessons for the five decades to come. Trans North Am. Wildl. Nat. Resour. Conf..

[12] Samuel MD, Storm DJ (2016). Chronic wasting disease in white-tailed deer: Infection, mortality, and implications for heterogeneous transmission. Ecology.

[13] Walters, B. F., Woodall, C. W. & Russell, M. B. *White-tailed deer density Estimates Across the Eastern United States, 2008*. Retrieved from the Data Repository for the University of Minnesota. 10.13020/D6G014 (2016).

[14] Michigan Department of Natural Resources (MDNR) and Michigan Department of Agriculture and Rural Development. *Michigan Surveillance and Response Plan for Chronic Wasting Disease (CWD) of Free-Ranging and Privately Owned Cervids. Report* (2012)

[15] Gneiting T, Raftery AE (2007). Strictly proper scoring rules, prediction, and estimation. J. Am. Stat. Assoc..

[16] R Core Team. R*: A Language and Environment for Statistical Computing* (R Foundation for Statistical Computing, 2018). http://www.R-project.org/.

[17] Russell RE, Katz RA, Richgels KLD, Walsh DP, Grant EHC (2017). A framework for modeling emerging diseases to inform management. Emerg. Infect. Dis..

[18] Cook, J. D., Walsh, D. P. & Hefley, T. J. *Bayesian Composition Sampling: U.S. Geological Survey Software Release*. 10.5066/P9XMF7FS (2023).

