# Supplementary material for: Bayesian forecasting of disease spread with little or no local data
Source: Sci Rep. 2023 May 19;13:8137. doi: 10.1038/s41598-023-35177-6 (PMC10199067; doi:10.1038/s41598-023-35177-6)
Supplement: Supplementary file 1 — Supplementary Information. [file 41598_2023_35177_MOESM1_ESM.docx]

**Appendix S1. Supporting Tables.**

Table S1.1 Parameter estimates and 95% Bayesian credible interval (CRI) for the spread and growth of CWD as reported in Hefley et al. (2017). Included are estimates for the intercept term (α) and landscape covariates of rivers, percent forest, and percent development in a 2.6 km^2^ grid cell.

| Spread Covariates | Median (α) | Lower 95% CRI | Upper 95% CRI |
| --- | --- | --- | --- |
| α_0_ | 1.72×10^1^ | 1.70×10^1^ | 1.74×10^1^ |
| River | 6.25×10^−1^ | 1.89×10^−1^ | 1.11 |
| Forest | −1.92×10^−1^ | −2.95×10^−1^ | −8.88×10^−2^ |
| Development | −1.30×10^−1^ | −3.44×10^−1^ | 1.40×10^−1^ |
| Growth Covariates | Median (γ) | Lower 95% CRI | Upper 95% CRI |
| γ_0_ | 6.91×10^−2^ | −1.76×10^−2^ | 1.43×10^−1^ |
| River | 2.19×10^−1^ | −4.03×10^−1^ | 8.01×10^−1^ |
| Forest | 2.09×10^−1^ | 1.20×10^−1^ | 3.21×10^−1^ |
| Development | 7.01×10^−2^ | −8.23×10^−2^ | 2.97×10^−1^ |

Table S1.2 Total number of individual white-tailed deer that were tested for chronic wasting disease from 2008–2016. 97.5% CRI (Bayesian credible interval) column is the potential adult male prevalence estimates that could exist in the population but remain undetected based on the total number of samples. Data from 12-township area (includes Cato, Douglass, Fairplain, Ferris, Maple Valley, Montcalm, Oakfield, Pine, Reynolds, Sidney, Spencer, Winfield) in Michigan U.S. where disease was discovered in 2017. Data source: Michigan Department of Natural Resources.

| Year | Fawn Male | Yearling  Male | Adult  Male | Fawn  Female | Yearling  Female | Adult  Female | Total | 97.5% CRI |
| --- | --- | --- | --- | --- | --- | --- | --- | --- |
| 2008 | 3 | 115 | 18 | 12 | 37 | 98 | 283 | 0.0356 |
| 2009 | 5 | 13 | 6 | 1 | 8 | 25 | 58 | 0.1556 |
| 2010 | 2 | 9 | 4 | 3 | 5 | 19 | 42 | 0.2089 |
| 2011 | 1 | 15 | 3 | 1 | 14 | 36 | 70 | 0.1345 |
| 2012 | 0 | 0 | 0 | 0 | 0 | 0 | 0 | — |
| 2013 | 0 | 0 | 0 | 0 | 0 | 0 | 0 | — |
| 2014 | 0 | 0 | 0 | 0 | 0 | 0 | 0 | — |
| 2015 | 0 | 2 | 1 | 0 | 3 | 0 | 6 | 0.7587 |
| 2016 | 0 | 6 | 1 | 0 | 7 | 11 | 25 | 0.3312 |
